# The impact of humidified high nasal flow oxygen in different groups of intensive care patients with respiratory failure

**DOI:** 10.1186/2197-425X-3-S1-A392

**Published:** 2015-10-01

**Authors:** FV Beers, D Ramnarain

**Affiliations:** Department Intensive Care, St Elisabeth Hospital, Tilburg, Netherlands

## Introduction

Recently, humidified high flow nasal cannula oxygen (HFNC) has gained popularity in treating patients with respiratory insufficiency. Studies have shown that HFNC generates a low level of positive airway pressure, reduction of airway resistance and flushes nasopharyngeal dead space leading to less work of breathing. We are using HFNC in a variety of patients since 2010.

## Objectives

We compared the outcomes of HFNC with conventional oxygen therapy in different groups of ICU patients with respiratory failure.

## Methods

We retrospectively studied respiratory -, oxygen derived- and hemodynamic parameters before and one hour after start of HFNC in 116 patients during 2010 and 2011. All patients were treated in a mixed medical, surgical, neurological ICU of a teaching hospital. We compared non-invasive oxygen therapy like venturi mask, non-rebreathing mask and non-invasive positive pressure ventilation with HFNC. The HFNC used an air-oxygen blender with adjustable Fi02 (0.21-1.0), delivering a modifiable gas flow of 40 l/min (Optiflow, Fisher&Paykel, Auckland, New Zeeland) in combination with humidification. Data from Metavision^TM^PDMS and Mediscore^TM^. We used oneway ANOVA to compare hemodynamic and oxygen related variables before and one hour after start of HFNC.

## Results

116 patients were included, 66 men and 50 women, mean age 66, SD 14, mean APACHE 4 on admission 64, SD 21. Mean duration of HFNC was 24, SD 31 hours.

Indications for HFNC could be divided in 6 categories; 1. Hypoxia: n = 41, 2. Weaning from NPPV: n = 25, 3. Comfort: n = 18, 4. No acceptance of non-invasive positive pressure ventilation (NPPV): n = 17, 5. Respiratory distress/comfort: n = 9, 6. Other: n = 6. In 21 patients we measured arterial blood gases (ABG). The oxygenation was significant better with HFNC, Pa02 (p = 0,019) and Pa02/Fi02 (p = 0,002). The PaC02 was significant lower (p = 0,048) with HFNC. In 116 patients the peripheral oxygen saturation (Sp02)/ fraction of inspired oxygen (Fi02) ratio was significant better (p = 0,000) with HFNC, the respiratory rate was significant lower (p = 0,000) with HFNC. Also the hemodynamic variables heart rate and mean arterial pressure were significant better with HFNC. Despite the use of HFNC, in 35 patients (30%) intubation was unavoidable and 29 patients (25%) died.

## Conclusions

We used HFNC therapy for different indications. Oxygen derived parameters significantly increased after one hour of HFNC. Also respiratory rate, heart rate and mean arterial pressure were significant better with HFNC compared with conventional oxygen therapy. HFNC was successful and well tolerated in patients weaning from NPPV. HFNC is a useful and a comfortable tool in oxygen therapy. There were no adverse effects of HFNC. Further research is necessary to identify which flow rate is the best in different indications of HFNC.

